# Supplementary material for: The interactome of intact mitochondria by cross-linking mass spectrometry provides evidence for coexisting respiratory supercomplexes
Source: Mol Cell Proteomics. 2017 Dec 8;17(2):216–32. doi: 10.1074/mcp.RA117.000470 (PMC5795388; doi:10.1074/mcp.RA117.000470)
Supplement: Supplemental Data [file supp_RA117.000470_133922_0_supp_23978_fzffwf.zip › spectra_annotation/mito_spectra_annotation/106-1-3-1-2-1.pdf]

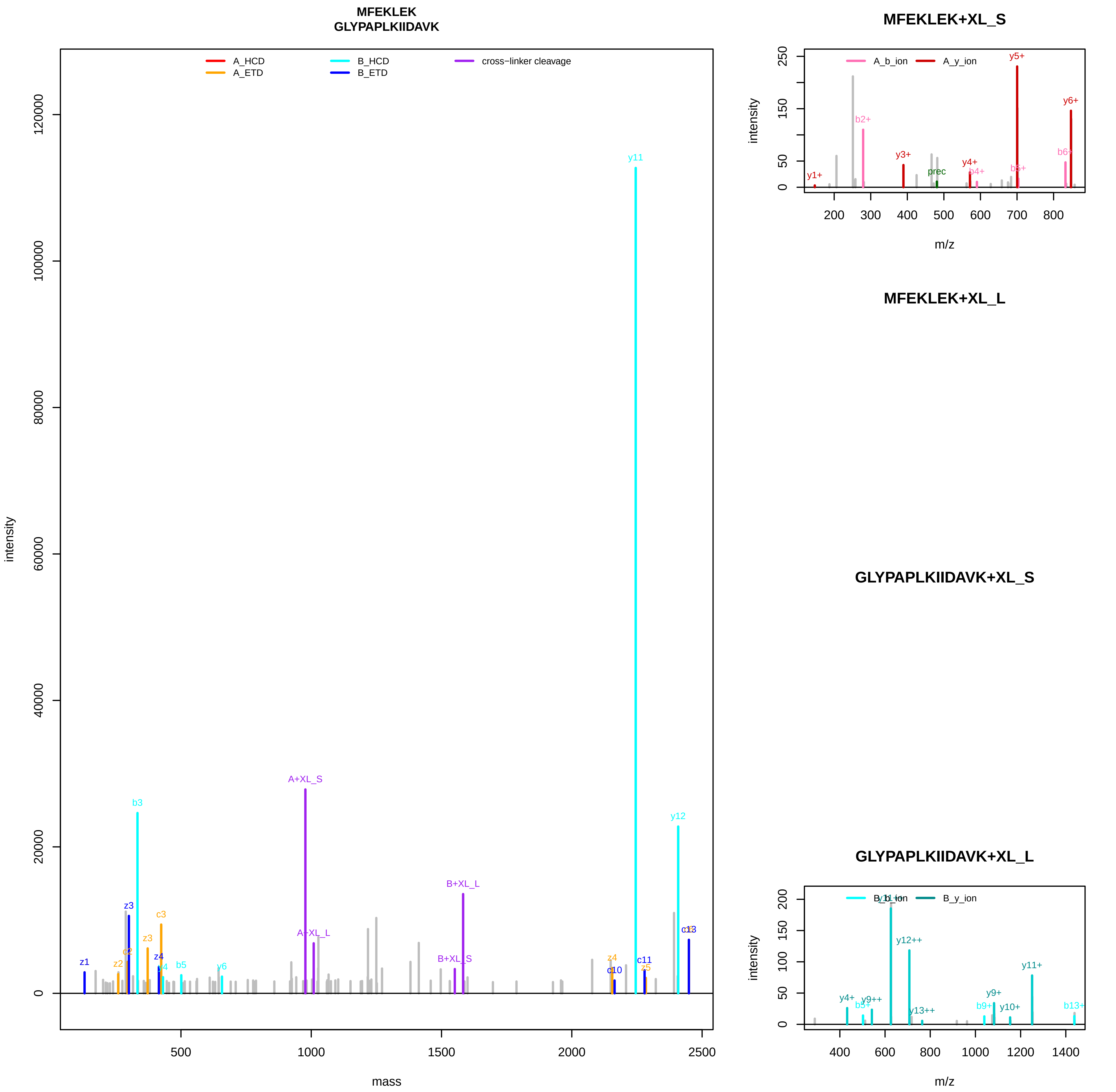

MFEKLEK
GLYPAPLKIIDAVK
A_HCD
A_ETD
B_HCD
B_ETD
cross−linker cleavage
intensity
mass
y11
y12
b3
A+XL_S
A+XL_L
B+XL_L
B+XL_S
c13
c11
c10
z5
z4
z1
z2
c2
z3
c3
y4
b5
y6
MFEKLEK+XL_S
A_b_ion
A_y_ion
y1+
b2+
y3+
prec
y4+
b4+
y5+
b5+
b6+
y6+
m/z
MFEKLEK+XL_L
GLYPAPLKIIDAVK+XL_S
GLYPAPLKIIDAVK+XL_L
B_b_ion
B_y_ion
y4+
b5+
y9++
y11++
y12++
y13++
b9+
y9+
y10+
y11+
b13+
